# Characterisation of human iPS cells harbouring the p.A329T variant of caspase-1

**DOI:** 10.1186/1546-0096-13-S1-P33

**Published:** 2015-09-28

**Authors:** J Thiem, A Rösen-Wolff

**Affiliations:** 1 University Hospital Carl Gustav Carus, Department of Pediatrics, Dresden, Germany

## Introduction

Naturally occurring genetic variants of the *CASP1* gene are associated with autoinflammation in patients suffering from recurrent febrile episodes and generalized inflammation. The resulting caspase-1 variants have reduced enzymatic activity due to destabilization of the caspase-1 tetramer. In addition, autoprocessing of the pro-caspase-1 variants is reduced and hence, CARD-CARD interactions with RIP2, enabling NFkB activation, is enhanced in a cell culture model.

## Objectives

In order to characterize the effects of the p.A329T caspase-1 variant detected in a child suffering from severe autoinflammatory symptoms, we tried to establish a reliable cell model based on patient derived induced pluripotent stem cells (iPSCs).

## Methods

We generated iPSCs by reprogramming fibroblasts of the patient's tympanic membrane (and wild type fibroblasts) using a replication deficient retrovirus (pRRL.PPT.SF.hOKSMco.idTomato.preFRT pMD.G (VSVG) psPAX2). We were able to verify the pluripotency of these iPSCs in different tests (quantitative real time PCR analysis of pluripotency markers, Immunocytochemistry of three germ layer markers and pluripotency markers). Subsequently the resulting iPSCs formed embryoid bodies (EB) which were then cultured in X-Vivo 15 media containing M-CSF and IL-3. After a few weeks, the adherent cells spreading from the settled EBs began to release monocytes (CD14+, CD45+, CD105+, CD192+). These were harvested from the supernatant and differentiated for 7 days in the presence of M-CSF (without IL-3). Thereafter, they were detached, counted and plated as 1x10^5^ cells per 96-well. After allowing them to rest for one day, we primed the cells with ultrapure LPS and stimulated them with ATP or Nigericin. The supernatant was analyzed for cytokine concentrations of IL-1β, IL-6, IL-10 and TNFα using BD™ Cytometric Bead Array.

## Results

The generated monocytes expressed the commonly established cell surface markers (CD14, CD16, CD45, CD163, and CD192) and could be differentiated into functional macrophages that seemed to react to stimulation as expected. Furthermore, first results indicated a different reaction of the cells generated from the patient (p.A329T) in comparison to the wild type control.

## Conclusion

Human induced pluripotent stem cells are a useful option to gain monocytes and macrophages in order to study defects of the immune system without the necessity to repeatedly draw large amounts of blood.

## Acknowledgements

This study was supported by German Network on Primary Immunodeficiency Diseases (pid.net).

